# Etoricoxib is safe and effective in preventing heterotopic ossification after primary total hip arthroplasty

**DOI:** 10.1186/s13018-021-02297-6

**Published:** 2021-02-27

**Authors:** Stella Oberberg, Jan Nottenkämper, Matthias Heukamp, Jan Krapp, Roland E. Willburger

**Affiliations:** Department of Orthopaedic Surgery, Katholisches Klinikum Bochum, Martin-Luther-Hospital, Voedestrasse 79, 44866 Bochum, Germany

**Keywords:** Etoricoxib, Undesirable side effects, Heterotopic ossification, Total hip arthroplasty

## Abstract

**Background:**

Heterotopic ossifications are a common complication after total hip arthroplasty. Low-dose radiation therapy and non-steroidal anti-inflammatory drugs have proven to effectively reduce the rate of heterotopic ossification after total hip arthroplasty. However, a low number of studies describe an equal efficiency of etoricoxib. This work shows first results on the examination of a larger group with 194 subjects to analyze efficiency and rate of side effects under treatment with etoricoxib.

**Methods:**

Clinical examinations were performed the day before surgery and after at least 12 months. The survey of clinical and functional outcome was done with Harris Hip Score (HHS). Conventional antero-posterior radiographs and second plane in frog leg position were assessed.

**Results:**

In total, 14 undesirable side effects (7.4%) and only four early terminations of therapy (2.1%) were documented. A complete 1-year follow-up examination including radiographs could be done in 143 subjects (79.4%). Only 28 subjects (19.6%) developed heterotopic ossifications from which 92.9% were classified in type 1 and 7.1% in type 2 using the method described by Brooker. The results do not show correlations with body mass index, extended treatment (more than ten days), or clinical and functional outcome (measured by “Harris Hip Score”). However, male subjects show a significantly higher rate of heterotopic ossifications.

**Conclusions:**

The investigations presented in this study confirm a good efficiency of etoricoxib for the prevention of heterotopic ossifications in comparison with classical methods such as radiation or drug therapy and show a low rate of undesirable side effects.

## Background

Heterotopic ossification (HO) is a well-known complication after total hip arthroplasty (THA). The origin and pathogenesis of HO are still unknown. The incidence of HO without sufficient prophylaxis varies from 8 to 90% after THA depending on the individual risk profile [1]. Up to 15% of all HO become symptomatic by causing pain and limited range of motion of the affected joint [2, 3]. In 2000, Eggli et al. [4] studied the effect of HO on the satisfaction rate of patients. They showed that severe HO after THA decreases the satisfaction rate to 30% compared to 90% for patients without HO. Low-dose radiation therapy [5] and non-steroidal anti-inflammatory drugs (NSAID) [1] have proven to effectively reduce the rate of HO after THA. However, the use of NSAIDs shows side effects such as gastrointestinal problems, prolonged bleeding time, and an increase of associated fractures [6]. In several studies up to 37% of patients receiving NSAID had to cease this medication due to undesirable side effects [7]. Selective cyclooxygenase (COX)-II blockers have a lower rate of gastrointestinal bleeding compared to classical NSAIDs and seem to be a reasonable alternative for oral prophylaxis with respect to contraindications like for example cardiovascular disorders [8]. Celecoxib, rofecoxib (withdrawn from the market), and etoricoxib have proven to reduce HO after primary THA [8–11]. The study of Winkler et al. [12] shows an equal efficiency of etoricoxib (90 mg once daily for nine days postoperative) compared to diclofenac (75 mg twice daily for nine days postoperatively) in preventing HO after primary cementless THA. Up to now, there are only two studies published concerning etoricoxib [11, 12], each with a small sample size (42 respectively 47 patients) and a limited follow-up time of 6 months. In the comment regarding the publication of Brunnekreef et al. [11], Zhang et al. [13] recommended a longer follow-up time of at least 1 year, a larger sample size, and special attention to adverse effects of etoricoxib. Winkler et al. [12] described 12.9% adverse events in the etoricoxib group related to the study medication compared to 9.4% in the diclofenac group. The most frequent adverse events in both groups were nausea, vomiting, dizziness, and diarrhea [12].

Our study describes first the results of a larger group of patients (194) getting etoricoxib to prevent HO after primary THA with a follow-up time of at least one year and a detailed verification of side effects and discontinuation rate due to side effects as demanded by Zhang et al. [13].

## Materials and methods

As COX-2-blockers are a common therapy to prevent HO after THA, the local ethical committee had no objection to this study. The study was conducted according to the Declaration of Helsinki on biomedical research involving human subjects. As a common method describing HO, we used the classification by Brooker et al. [14]. This classification differs in four grades of HO using radiographs of the hip in two planes. Grade 1 means there are few islands of bone in the soft tissue, and grades 2 and 3 mean bone spurs between pelvis and femur leaving at least 1 cm (grade 2) or less than 1 cm space (grade 3) between bones. Grade 4 describes a bony ankylosis of the hip. Hip function was measured using Harris Hip Score (HHS) [15]. The score rates the categories “pain” (44/100 points), “function” (47/100 points), and “range of motion/deformity” (nine/100 points). For calculation of the category “range of motion/deformity,” we used the simplified method described by Haddad et al. [16].

### Statistics

Mean values and percentages were calculated. Mann-Whitney *U* test was used to determine if there is a connection between the occurrence of HO and the result of HHS. Fisher’s exact test was used to determine if there is a correlation of body mass index (BMI), extended treatment (more than ten days), clinical and functional outcome (measured by “Harris-Hip-Score”), and gender with the occurrence of HO. Odds ratio (95%) confidence interval was calculated and tested with Fisher’s exact test if diagnoses and occurrence of HOs were independent of each other.

### Patients

All patients in our department who had primary cementless THA in 1 year (204 consecutive patients) were theoretically due to be included in this study. Exclusion criteria (EC) were previous allergic reaction on etoricoxib, a history of gastrointestinal ulcers or perforations, hepatic dysfunction, renal dysfunction with a clearance below 30 ml/min, and cardiac insufficiency. Most patients included had a primary osteoarthritis (79%), 8% a secondary osteoarthritis (dysplasia, condition after proximal femur fracture, condition after femoral change osteotomy), 8% a femoral head necrosis, and 5% an inflammatory joint disease like rheumatoid arthritis or peripheral spondylarthritis. Mean age at surgery was 70 years (35–87 years, median 71.5 years). We examined 204 subjects (142 females, 62 males).

### Pain medication

The administration of NSAID was ceased before surgery; if necessary, novalgin (metamizol) and/or tilidin (naloxon hydrochlorid) were allowed to alleviate pain. The morning after surgery, patients received their first dose of etoricoxib (120 mg orally for 3 days) and afterwards 90 mg once daily and continued at least for 10 days. Patients were not allowed to use any other NSAID. If patients used medication for gastrointestinal complaints prior to surgery (like pantoprazol), they were allowed to continue this medication.

### Surgery and rehabilitation

In all patients, a lateral transgluteal approach (described by Bauer) was used and a cementless total hip prosthesis was implanted (HI screw cup, Polarstem, Smith & Nephew). At the beginning of the surgery, patients received a single dose antibiotic prophylaxis (cefazolin, 2 g intravenously). Suction wound drainages (intra-articular and subcutaneous) were used for 2 days. The first day after surgery full-weight bearing was allowed with two elbow crutches. Rehabilitation was started in the hospital and continued in a follow-up treatment in an inpatient or outpatient rehabilitation facility.

### Clinical examination and questioning

Clinical examinations were performed the day before surgery, at discharge from the hospital and after at least 12 months. The survey of clinical and functional outcome was done with Harris Hip Score (HHS). Conventional antero-posterior radiographs and second plane in frog leg position (Lauenstein method) were assessed. HOs were classified using the method described by Brooker et al. [14]. Radiographs were evaluated by the third and last author. In case of uncertainties, radiographs were evaluated by an experienced radiologist of our hospital.

## Results

See Tables 1, 2, 3, and 4 for the results.

**Table 1:** Sample size: all subjects with primary total hip replacement

	Preop
**Theoretical due**	**204**
Excluded because of EC against etoricoxib	4 (A)
Excluded because of PAO (periarticular ossifications) prophylaxis with different NSAID	6 (B)
Included	194

(A) Two patients with strict contraindication against NASIDs due to cardiac preexisting disease both received radiation therapy (once seven Gray). Two patients with history of stroke rejected both prophylactic options (radiation and etoricoxib or any NSAID). (B) Three patients preferred expressly prophylaxis with diclofenac, another three patients with ibuprofen

**Table 2:** Follow-up data: all subjects with etoricoxib for PAO prophylaxis

Follow-up etoricoxib	Preop	Adherence to therapy (10 days)	Undesirable side effects	Early termination of therapy
Included	194			
Clinical examination at discharge	194	190 (97.9%)	14 (C) (7.4%)	4 (D) (2.1%)

(C) Five patients reported gastric complaints that disappeared under symptomatic therapy with a proton pump inhibitor. Two patients had slightly increased blood pressure (not in need of therapy). Two patients reported cardiac arrhythmia that could not be detected with an electrocardiogram. One patient developed increased kidney retention values which returned to normal after stopping the drug (etoricoxib). Three patients reported fatigue. One patient showed a skin reaction with itching and rash that disappeared after weaning and local as well as oral administration of an antihistamine. (D) The administration of etoricoxib was early terminated in the two patients reporting cardiac arrhythmia, in the patient with increased kidney retention values, and the patient with skin reaction

**Table 3:** Follow-up data 1 year postop

Follow-up PAO	Preop	Follow-up at least 1 year postop.
Included	194	
Deaths	0	3 (E)
Excluded because of early stop of PAO prophylaxis	0	4 (D, see above)
Excluded because of joint dislocation or revision surgery	0	7 (F)
**Expected**	**194**	**180 (G)**
Actual complete examination including radiographs	194	143 (H) (79.4%)
Clinical examination but no radiographs at follow-up	0	14 (I) (7.8%)
Only contact by phone	0	22 (J) (12.2%)
Not available	0	1 (K)

(E) All three patients died more than 6 months after surgery because of carcinoma already known preoperatively (pharynx, prostate, breast); death was unrelated to surgery or PAO prophylaxis. (F) One patient suffered dislocation of the hip 4 months postoperatively (closed reposition of the hip). Revision surgery was performed in five patients: Two periprosthetic infections (diagnosed 19 respectively 28 days postoperatively) with two stage implant exchange, two wound healing disorders without proof of germs, one cup dislocation with component exchange 11 months postoperatively. One revision surgery because of traumatic periprosthetic fracture 9 months postoperatively. (G) Expected = Included minus deaths, minus excluded for early stop of PAO prophylaxis, minus joint dislocation or revision surgery. (H) Actual complete examination including radiographs at follow-up = maximum number of subjects for data analysis concerning periarticular ossification (=100%). (I) Patients did not agree to any x-ray control; everyone was satisfied with the result of the operation. (J) Patients were not ready for a follow-up visit (reduced mobility due to age, great distance to the hospital, said they had no time); everyone was satisfied with the result of the operation. (K) Patient moved to unknown location

**Table 4:** All subjects with radiographs at least 12 months postoperatively

Complete examination including radiographs	143 (99 females, 44 males)
Brooker 0, no HOs	115 (80.4%)
Brooker 1	26
Brooker 2	2
Brooker 3	0
Brooker 4	0

In at least two subjects (hip dislocation 4 months postoperatively, traumatic fracture 9 months postoperatively) the complication was unrelated to the PAO prophylaxis with etoricoxib. In the remaining five patients (periprosthetic infections, wound healing disorders, cup dislocation), an influence is possible but unlikely.

No subject showed HOs Brooker grade 3 or 4. HOs were found more frequently in men (15 males, 13 females). There was no significant association between the occurrence of HOs and preoperative diagnosis, BMI (body mass index), and result of HHS.

## Discussion

Our investigation shows that etoricoxib is safe and effective in preventing HOs after total hip replacement. Meta-analyses on randomized clinical trials showed that selective COX-2 Inhibitors and non-selective NSAIDs like diclofenac and indomethacin are equally effective in the prevention of HO after THA [8]. We confirm that a period of 10 days taking etoricoxib is sufficient to reduce the rate of HOs. Therefore, side effects based on extended administration of the drug can be avoided respectively reduced. In comparison with Moed and Maxey [17], we found a higher incidence of HOs for male subjects [17]. As no subject showed HOs Brooker grade 3 o 4 no relevant limitation of joint mobility had to be estimated. Most studies in the literature did not report undesired side effects in detail or the abandonment rate as a result of side effects. Cella et al. [7] reported that 37% of the patients that used indomethacin had to cease medication because of side effects. Jockheck et al. [18] reported stop of medication in 15.5% of their patients getting diclofenac for prophylaxis. Such a high abandonment rate is not tolerable as we know that a perioperative radiation is only effective when performed in a time window of 20 h before and 96 h after surgery (optimal effect 8 h before and 72 h after surgery) [1]. In many cases, the oral prophylaxis is terminated later due to side effects and the patient is then outside of this optimal time window for radiation. Thus, an early terminated oral prophylaxis can often not be replaced by a delayed radiation. Consequently, an oral HO prophylaxis should be safe and as reliable as possible in terms of adherence to therapy. In our study, only 2.1% ceased the treatment because of side effects.

Based on our results and the literature, we can summarize that etoricoxib is a very good alternative for oral HO prophylaxis with a low rate of side effects and high adherence to therapy. To increase the validity of the study, it would be useful to add a study including a control group.

## Data Availability

All authors declare that all data and materials support the published claims and comply with field standards.

## Abbreviations

HO: Heterotopic ossification
THA: Total hip arthroplasty
NSAID: Non-steroidal anti-inflammatory drugs
COX: Selective cyclooxygenase II blockers
HHS: Harris Hip Score
EC: Exclusion criteria
PAO: Periarticular ossifications
